# Enhancing Hand Fracture Care: A Prospective Study of Artificial Intelligence Application With ChatGPT

**DOI:** 10.1016/j.jhsg.2024.03.014

**Published:** 2024-04-30

**Authors:** Connor John Atkinson, Ishith Seth, Marc Adam Seifman, Warren Matthew Rozen, Roberto Cuomo

**Affiliations:** ∗ Department of Plastic and Reconstructive Surgery, Frankston Hospital, Peninsula Health, Frankston, VIC, Australia; † Department of Surgery, Central Clinical School, Monash University, Alfred Hospital, Prahran, VIC, Australia; ‡ Plastic Surgery Unit, Department of Medicine, Surgery and Neuroscience, University of Siena, Siena, Italy

**Keywords:** Artificial intelligence, ChatGPT, Hand fracture, Large language model, Management

## Abstract

**Purpose:**

The integration of artificial intelligence and machine learning technologies into the medical field has brought about remarkable advancements, particularly in the domain of clinical decision support systems. However, it is uncertain how they will perform as clinical decision-makers.

**Methods:**

This prospective cohort study evaluates the potential of incorporating ChatGPT-4 plus into the management of subcapital fifth metacarpal fractures. The treatment recommendations provided by ChatGPT-4 plus were compared with those of the two control groups—the attending clinic plastic surgeon and an independent expert panel. The primary outcome measures, operative or conservative, were compared between the groups. Intraclass correlation of 0.61 infers moderate reliability in the consistency of recommended management plans across all groups.

**Results:**

Key predictors for opting for operative management, regardless of the decision-maker, included clinical signs of scissoring, extension deficit, and radiographic evidence of intra-articular extension.

**Conclusions:**

These findings support the potential for artificial intelligence applications in enhancing diagnostic and treatment decisions.

**Type of study/level of evidence:**

Therapeutic IV.

Interest has recently surged in employing artificial intelligence (AI), specifically large language models (LLMs) like ChatGPT, to augment health care processes and act as clinical decision support systems.^1^, ^2^, ^3^ ChatGPT leverages natural language processing and machine learning and has the potential to aid clinicians in decision making by offering rapid access to the extensive information within its training data. The application of LLMs in surgery and surgical research is far-reaching,^4^, ^5^, ^6^, ^7^, ^8^, ^9^ although more focused studies on its direct application in managing surgical conditions, notably hand fractures, are limited.^10^

Subcapital fifth metacarpal fractures, also known as boxer’s fractures—given their association with the mechanism of injury, are common injuries accounting for 10% of all hand fractures. These fractures are most commonly observed in males with peak incidence between 10 and 29 years.^11^ The majority of these fractures can be managed conservatively with ulnar gutter immobilization, especially those that are uncomplicated, closed, nonangulated, and nonmalrotated. However, there is debate surrounding the optimal treatment of fractures with less favorable characteristics, with operative management typically reserved for those with a clear indication.^12^

The natural kinematics of a clenched fist suffering axial loading force typically results in palmar angulation as a result of intrinsic and extrinsic muscle action.^13^ The biomechanical sequelae of such can result in decreased grip strength and range of motion, especially in fractures exceeding 30° volar angulation.^13^, ^14^, ^15^ Inappropriate treatment can result in biomechanical disadvantage; thus, choosing the optimal therapy for each patient is essential to preserve maximal hand function.^16^ This decision should take into account the individual’s professional needs and recreational activities to ensure a treatment plan that is both effective and conducive to their lifestyle.

Immediate immobilization of fractures with up to 70° of volar angulation without rotational deformity has been appropriately managed with a pressure bandage and immediate mobilization, without comparable functional disadvantage. Although in these circumstances, patients should be willing to accept cosmetic compromise, with loss of knuckle projection.^17^^,^^18^ This exemplifies the variability in approach to management and proved to be an ideal platform for our study, where multiple patient and fracture factors must be considered in tandem to optimize patient outcomes.

Important clinical and radiological variables when considering a patient for operative management include fracture pattern, presence of intra-articular extension, displacement of distal fragment, metacarpal shortening >5mm, and rotational deformity.^12^^,^^19^^,^^20^

To the best of our knowledge, this is the first prospective cohort study investigating the application of an LLM in formulating surgical management for patient care. The potential ability to harness machine learning and LLMs to generate appropriate management plans for hand fractures could have broader implications across health care. It is therefore vital to assess the accuracy and dependability of the information and management plans formulated and further recognize AI-generated response limitations, such as contextual information scarcity and algorithm biases.

## Methods

**Figure 1:**
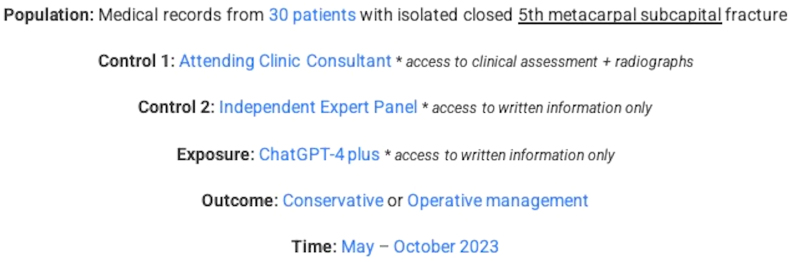
PECOT diagram of study design.

This prospective cohort study, in accordance with strengthening the reporting of observational studies in epidemiology guidelines,^21^ aims to evaluate the potential of ChatGPT-4 plus in managing hand fractures. Conducted at a single plastic surgery outpatient clinic in Melbourne from May to October 2023, the study included 30 patients with isolated subcapital fifth metacarpal fractures (Fig. 1). The primary objective was to evaluate the appropriateness of the treatment plan formulated by ChatGPT-4 plus (ie, similar to that of a trained clinician) when the AI system is presented with objective clinical and radiological findings. This was done by prospectively comparing the treatment plan devised to that of two control groups—an independent expert panel and an attending outpatient clinic consultant. The hypothesis suggested that ChatGPT-4 plus could provide management plans on par with medical professionals.

The study did not involve any active intervention, with all patients following the recommended treatment plan from the attending outpatient clinic consultant. This group is defined as the primary control group and has the ability to independently assess each patient and review their radiographs. The secondary control group is defined as the independent expert panel formed by two board-certified plastic surgeons (M.A.S. and W.M.R.). This group was blinded and provided with the same written information on each patient as the exposure group, defined at ChatGPT 4 plus, to ensure a better control comparison. These groups could not directly assess the patient or access radiographic images.

Inclusion criteria encompassed newly referred patients to the plastic surgery outpatient department with an isolated fifth metacarpal subcapital fracture, excluding any open fractures. Data were collected prospectively from various sources, including patient notes, radiographic imaging, and verbally from with surgical colleagues and hand therapists to ensure a complete data set. Radiological findings were standardized by the primary investigator, who independently interpreted all radiographs and provided objective information on each case.

**Figure 2:**
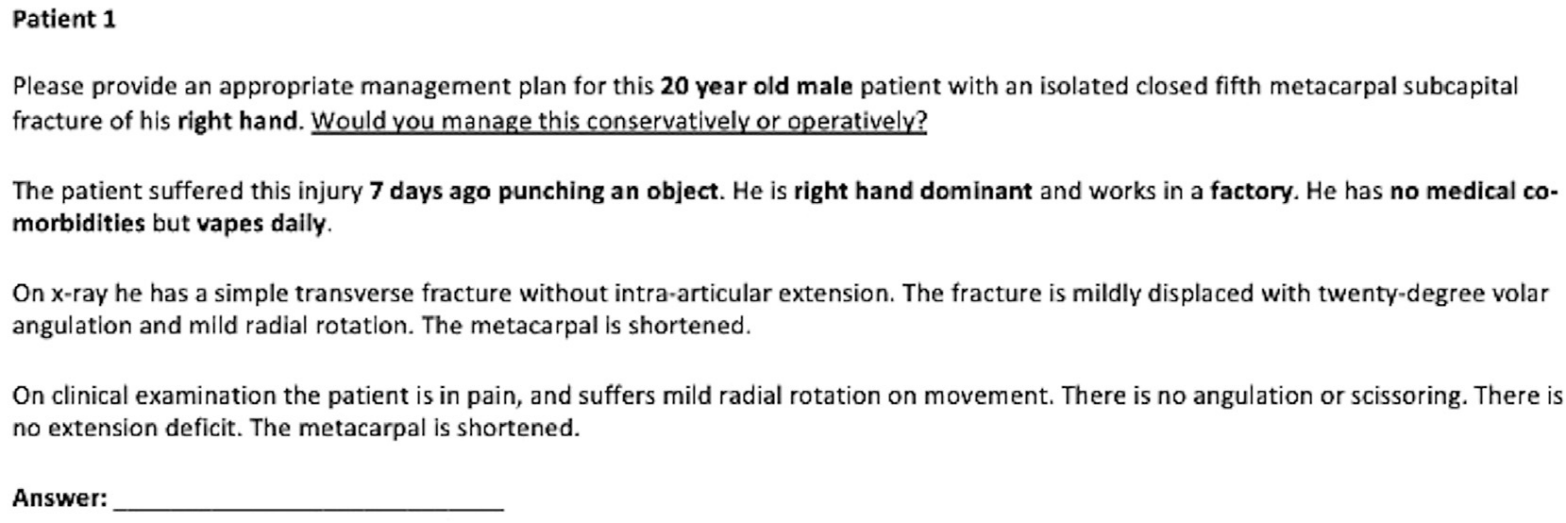
Written prose of study ID patient one, provided to the secondary control group and exposure group.

Data were subsequently deidentified, with each participant allocated a study ID and maintained on a password-protected database. Detailed written reports, including all clinical and radiological findings, were prepared for each patient (Fig. 2) and provided to the independent expert panel and ChatGPT-4 plus for evaluation.

Descriptive statistics summarize demographic, radiological, and clinical characteristics. Continuous variables are expressed as means and SD, whereas categorical variables are presented as frequencies and percentages. The primary outcome assesses the management plans formulated by the exposure group for both control groups. Comparisons between treatment plans for each patient utilize intraclass correlation. Any factors identified as possible predictors (demographic, clinical, and radiologic characteristics) were investigated using logistic regression analysis. A *P* value of < .05 was considered statistically significant.

## Results

Table 1 summarizes the patient characteristics of our cohort and is consistent with the literature, providing our study a reliable platform to work from. The median age is 20.5 years old, and most commonly right hand-dominant males suffer right-sided fractures as a result of punching mechanisms. These data also highlight that most patients injured their dominant hand, an important consideration when determining management plans.

**Table 1:** Descriptive Statistics of Patient Characteristics

Patients Characteristics		
**Age years median (IQR)**		20.5 (21)
**Male**		83%
**Hand dominance**	*RHD*	93%
	*LHD*	7%
	*Injured dominant hand*	87%
**Occupation**	*Sedentary*	63%
	*Manual*	37%
**Has medical comorbidities**		33%
**Smoker**		30%
**Fracture site**	*Left fifth MC SC*	13%
	*Right fifth MC SC*	87%
**Time from injury to assessment days median (IQR)**		7 (5)
**Mechanism of injury**	*Punch*	53%
	*FOOSH*	33%
	*Blunt*	10%
	*Crush*	3%

FOOSH, fall on an outstretched hand; LHD, left hand dominance; RHD, right hand dominance.

The majority of patients have also been assessed in the outpatient clinic 7 days postinjury, with only three patients seen beyond the interquartile range at 18, 22, and 42 days, respectively. These cases were the result of delayed presentation and subsequent referral. These patients were managed conservatively by both control and exposure groups, except one at 22 days, for which the AI recommended operative intervention and specialist review, given the accompanying mild radial rotation and the potential impact on the patient's occupation as a tiler.

Table 2 summarizes the treatment frequency outcomes for both the control and the exposure groups. A difference in operative frequency exists between groups that are independently assessing the patient and radiographs (ie, the primary control group) and the other groups formulating a plan based on the written prose. With the former trending to a more conservative approach.

**Table 2:** Treatment Frequency Outcomes of Control and Exposure Groups

Outcomes	Conservative	Operative
Control 1: Clinic consultant	28/30 (93%)	2/30 (7%)
Control 2: Expert panel	25/30 (83%)	5/30 (17%)
Exposure: ChatGPT-4 plus	25/30 (83%)	5/30 (17%)

Only two patients were managed operatively in real clinical practice. The independent expert panel not only agreed with these two interventions but also went on to theoretically offer three additional patients’ operative interventions. ChatGPT-4 plus also offered five operative interventions, although these were not always the same. The correlation between groups is better assessed using intraclass correlation. An intraclass correlation value of 0.61 confirms moderate reliability in the consistency of treatment plans across all groups as seen in Table 3.

**Table 3:** Intraclass Correlation Model

ICC	95% CI	
	Lower limit	Upper limit
**0.61**	0.28	0.8

Bold values indicate statistical significance (*P* < .05).

When looking at independent factors as predictors of treatment, we found a statistically significant association (*P* = .01) between having a medical comorbidity and the choice of operative intervention for all three groups (Table 4). However, there was no statistically significant interaction between having medical comorbidity and the type of decision-maker, inferring this factor is a predictor that influences decisions from all three groups.

**Table 4:** Patient Factors as Predictors of Treatment

Predictor	OR	*P* Value	95% CI	
			Lower Limit	Upper Limit
*Decision-maker*	1	1.0	0.46	2.2
*Medical comorbidity*	5.6	.01	1.5	21.3
*Smoker*	1.5	.6	0.4	6.7
*Time since injury days*	1.0	.8	0.9	1.1
*Dominant hand injured*	1.4	.8	0.14	13.6

There was a statistically significant association between having scissoring (*P* = .02) and extension deficit (*P* = .004) and the choice of operative intervention. Again, there was no statistically significant interaction between scissoring or degree of extension deficit and the decision-maker (Table 5).

**Table 5:** Clinical Assessment Factors as Predictors of Treatment

Predictor	OR	*P* Value	95% CI	
			Lower Limit	Upper Limit
*Decider*	1	1	0.45	2.2
*Pain*	2	.5	0.3	11.6
*Digit rotation*	0.9	.86	0.14	5.2
*Scissoring*	8.1	.02	1.3	49.9
*Metacarpal shortening*	1.7	.55	0.3	9.8
*Extension deficit*	1.1	.004	1.0	1.2

There was a statistically significant association (*P* = .02) between having intra-articular extension and the choice of operative intervention. Once again, there was no statistically significant interaction between intra-articular extension and the decision-maker (Table 6).

**Table 6:** Radiological Assessment Factors as Predictors of Treatment

Predictor	OR	*P* Value	95% CI	
			Lower Limit	Upper Limit
*Decision-maker*	1	1.0	0.38	2.6
*Intra-articular extension*	34	.02	1.7	664
*Angulation degree*	1.1	.1	0.98	1.22
*Rotation*	3	.3	0.34	29.9
*Metacarpal shortening*	3	.2	0.5	18.5

## Discussion

This comprehensive study offers preliminary evidence that an LLM like ChatGPT-4 plus is capable of formulating appropriate management plans for subcapital fifth metacarpal fractures. These plans show a moderate correlation with those devised by medical professionals, as indicated by the intraclass correlation. This consistency between ChatGPT and practitioners has been similarly observed in the management of head and neck cases in a recent study by Lechien et al.^22^ However, further research with larger cohorts, randomized controlled trials, and evaluations of patient outcomes following AI-assisted management is crucial for the robust validation of AI performance in surgical management.

**Figure 3:**
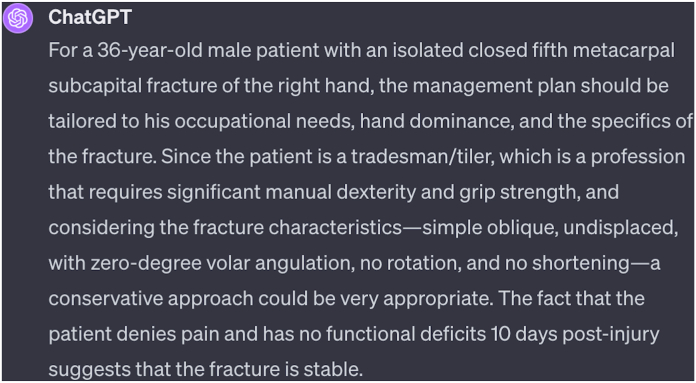
ChatGPT-4 plus treatment recommendation for study ID patient eleven.

This study reassuringly demonstrates ChatGPT’s concordance with both control groups in identifying independent predictors for treatment decisions, irrespective of the decision-maker. Such factors include medical comorbidities, scissoring, extension deficit, and intra-articular extension, demonstrating ChatGPT’s capacity to consider a range of factors in its recommendations, as do clinicians. This finding suggests that ChatGPT-4 plus can integrate clinical and radiological variables into its decision-making process, rather than basing choices solely on fracture characteristics. Anecdotally, we can further comment on the AI’s active consideration of each patient’s occupation and hand dominance when deliberating the optimal recommendation (Fig. 3).

Another notable finding was the difference in the frequency of operative recommendations between the group that independently assessed patients (primary control) and those that formulated management plans based on written prose (secondary control and exposure groups). The primary control group trended toward fewer operative interventions, indicating an underlying importance of surgeon–patient dynamic in the decision-making process. Comparison between the AI system and the primary control group was limited, as we were unable to control for independent clinician assessment and understanding of patient preference. However, the rapid advancement of AI now encompassing the interpretation and synthesis of different input modalities beyond written text, such as audio and visual images, could help overcome these limitations. The well-established integration of AI in radiology and its accuracy in radiograph interpretation are particularly noteworthy.^23^, ^24^, ^25^, ^26^, ^27^ The developing role of audio input not only facilitates dialog between the user, clinician, and patient but also complements ChatGPT’s advanced natural language processing capabilities, potentially enhancing the simulation of the surgeon–patient relationship.

Although this study provides valuable insights into the potential integration of ChatGPT-4 plus in managing subcapital fifth metacarpal fractures, several limitations warrant consideration. First, the relatively short follow-up period restricts our understanding of the long-term efficacy and outcomes of ChatGPT-assisted management. Longitudinal studies with extended follow-up are necessary to evaluate the sustained impact and utility of AI in clinical decision making. Conducting the study in a single center may also limit the generalizability of the findings. Diverse clinical settings and patient populations might yield different results; thus, multicenter studies are essential to validate and understand the broader implications of ChatGPT in various health care environments. Another critical limitation is the potential for confirmation bias. ChatGPT’s recommendations are based solely on the information provided by clinicians, which might vary significantly between a surgeon and a triage nurse, for example. This variability could influence the AI’s decision-making process and the resulting management plans. Regarding the AI itself, ChatGPT is limited by its training data, the specifics of which (such as the textbooks, journals, and websites used) are not publicly disclosed. This lack of transparency raises concerns about the comprehensiveness and biases of the underlying data. If the model uses journal articles, there is a risk of incorporating journal-specific biases into its recommendations. Additionally, like all AI systems, ChatGPT is susceptible to algorithmic biases, which can inadvertently affect its decision-making process. Ethical considerations also play a significant role in the deployment of AI in clinical settings. Ultimate responsibility for patient care must rest with the treating clinician or department, not the AI. There are also governance issues to consider, such as third-party access to patient information input into the software, which could have implications for patient privacy and data security.

## Conclusion

This novel investigation marks a pivotal step in understanding AI’s role in health care, focusing on ChatGPT-4 plus’s potential to manage subcapital fifth metacarpal fractures. The results revealed favorable outcomes, with a notable consistency between the recommendations made by ChatGPT-4 plus and experienced plastic surgeons. However, the study also brings to light the limitations of AI, emphasizing the importance of the surgeon–patient dynamic and suggesting that AI should complement, rather than replace, clinical judgment. The promising findings of this study advocate for further research to explore the potential application of AI in diagnostic and management decisions across various fields, beyond just hand fractures. Future studies, including large-scale, multicenter trials and long-term follow-up evaluations, are essential to validate and expand upon these initial findings, ultimately paving the way for AI to revolutionize health care and patient management.

## Conflicts of Interest

No benefits in any form have been received or will be received related directly to this article.
